# Improving the stability of photodoped metal oxide nanocrystals with electron donating graphene quantum dots†

**DOI:** 10.1039/d3nr03534d

**Published:** 2023-09-30

**Authors:** Andrea Camellini, Luca Rebecchi, Andrea Rubino, Wenhui Niu, Sang Won Kim, Ji Ma, Xinliang Feng, Ilka Kriegel

**Affiliations:** a Functional Nanosystems, Istituto Italiano di Tecnologia (IIT) via Morego 30 16163 Genova Italy Andrea.Camellini@iit.it Ilka.Kriegel@iit.it; b Department of Mechanical Engineering, Columbia University New York New York 10027 USA; c Dipartimento di Chimica e Chimica Industriale, Università degli Studi di Genova Via Dodecaneso 31 16146 Genova Italy; d Center for Advancing Electronics Dresden (cfaed) & Faculty of Chemistry and Food Chemistry, Technische Universität Dresden 01062 Dresden Germany; e Max Planck Institute of Microstructure Physics Weinberg 2 06120 Halle Germany; f Samsung Advanced Institute of Technology (SAIT), Samsung Electronics Co. Ltd Suwon 16678 Republic of Korea

## Abstract

Doped metal oxide nanocrystals are emerging as versatile multi-functional materials with the potential to address several limitations of the current light-driven energy storage technology thanks to their unique ability to accumulate a large number of free electrons upon UV light exposure. The combination of these nanocrystals with a properly designed hole collector could lead to steady-state electron and hole accumulation, thus disclosing the possibility for light-driven energy storage in a single set of nanomaterials. In this framework, it is important to understand the role of the hole collector during UV light exposure. Here we show, *via* optical absorbance measurements under UV light, that well-defined graphene quantum dots with electron-donating character can act as hole acceptors and improve the stability of the photo-generated electrons in Sn-doped In_2_O_3_ nanocrystals. The results of this study offer new insight into the implementation of photo-charged storage devices based on hybrid organic/inorganic nanostructures.

## Abstract 2

Functionalization of Sn-doped In_2_O_3_ NCs with specifically designed electron-donating graphene quantum dots (HBC–AOM) enhances the stability of photodoped electrons over prolonged UV light exposure.

## Graphical abstract

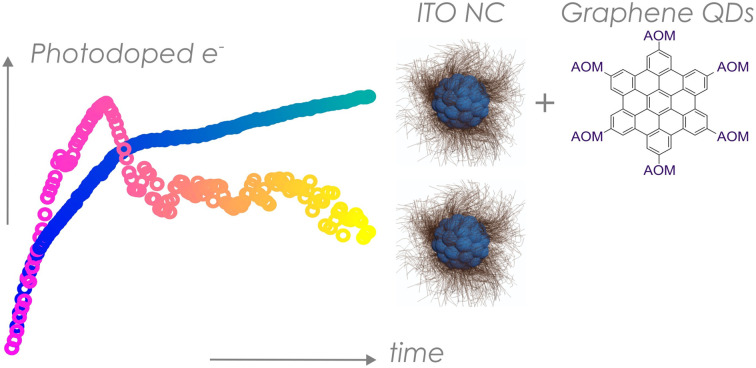

## 1. Introduction

The need to overcome the current technological limitations in solar energy conversion and storage^1^ calls for innovative approaches toward integration into a single monolithic device. To this end, doped metal oxide nanocrystals (NCs) are emerging as light-driven electron collectors due to their ability to store multiple delocalized electrons in the conduction band upon ultraviolet (UV) light illumination.^2^ This latter mechanism known as photodoping or photo-charging has been thoroughly investigated in standard photocatalytic metal oxide NCs such as ZnO^3^ and TiO_2_,^4^ as well as Sn-doped In_2_O_3_ (ITO),^5,6^ and ITO-core/In_2_O_3_-shell^7,8^ NCs. In brief, the material is irradiated with above-bandgap light under anaerobic conditions and in the presence of a sacrificial molecule (*e.g.* ethanol, EtOH). The latter is able to quench the photo-excited holes, leaving the NCs with a net excess of photo-excited electrons in the conduction band. Local electrical neutrality is then maintained by either the formation of a double layer at the surface of the NCs or by the intercalation of a charge-compensating ion (H^+^ cations) into the NC matrix.^9^

The possibility to effectively utilize the photogenerated holes in order to achieve steady-state photo-driven electron–hole storage, although so far highly overlooked, was recently demonstrated by replacing the sacrificial hole quencher with an efficient hole acceptor in close contact with the photodoped NCs.^10^ In this context, one of the limiting factors for the establishment of steady-state electron–hole separation is represented by the temporal stability of the photodoped electrons to prolonged UV exposure, in the presence of the hole accepting agent. The aim of this work is to address the long-term stability issue concerning the photodoped electrons in ITO nanocrystals by means of specifically designed graphene quantum dots (GQDs).^11^ The combination of these two classes of nanomaterials allows for an improvement in the stability of the ITO excited charges over longer UV irradiation time, thanks to the GQDs’ ability to permanently delocalize holes in their highest occupied molecular orbital (HOMO) level. Specifically, here we study the stability of photodoped electrons in a functionalized mixture of ITO NCs and electron-donating HBC–AOM graphene quantum dots, in which the hexa-*peri*-hexabenzocoronene (HBC)^12,13^ has been decorated with the functional anthracenyl units and *N*-octylmaleimide (AOM) groups.

## 2. Materials & methods

### 2.1. Synthesis of HBC–AOM graphene quantum dots

**Scheme 1:**
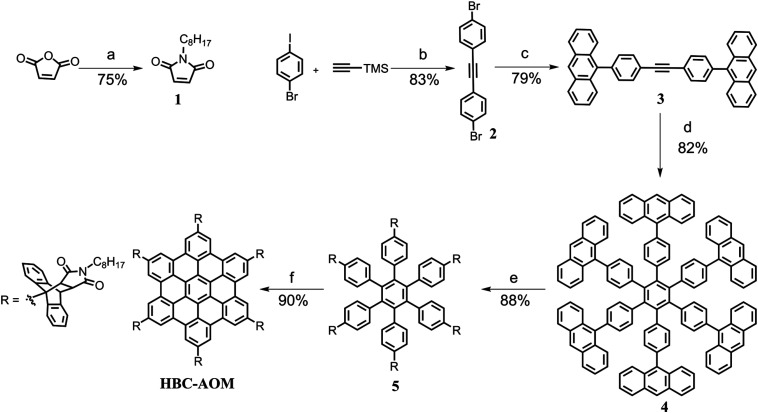
Synthetic route towards HBC–AOM. (a) Octylamine, ZnBr_2_, hexamethyl disilazane, toluene, 115 °C, and 2 h. (b) PdCl_2_(PPh_3_)_2_, CuI, DBU, toluene/H_2_O, room temperature, and 18 h. (c) Pd(PPh_3_)_4_, K_2_CO_3_, 9-anthraceneboronic acid, dioxane/H_2_O, 100 °C, and overnight. (d) Co_2_(CO)_8_, dioxane, 110 °C, and 14 h. (e) *N*-Octylmaleimide **1**, *o*-xylene, 150 °C, and 48 h. (f) FeCl_3_, CH_3_NO_2_, CH_2_Cl_2_, room temperature, and 5 h.

The synthetic route towards HBC–AOM is shown in Scheme 1. Firstly, *N*-octylmaleimide 1 was synthesized from maleic anhydride in 75% yield. Then, 1,2-bis(4-bromophenyl)ethyne 2 was obtained in 83% yield by a Sonogashira coupling reaction between the commercially available 1-bromo-4-iodobenzene and trimethylsilylacetylene. After that, a Suzuki coupling reaction between 2 and 9-anthraceneboronic acid afforded 1,2-bis(4-(anthracen-9-yl)phenyl)ethyne 3 in 79% yield. Subsequently, the cobalt-catalyzed cyclotrimerization of 3 gave the key intermediate 4 in 82% yield, followed by the Diels–Alder cycloaddition with 1 giving compound 5 in 88% yield. Finally, HBC–AOM was achieved with a yield of 90% through the FeCl_3_-mediated Scholl reaction of 5. The synthesis of 1 ^14^ and 2 ^15^ was performed in accordance with the literature protocol. A detailed description of the synthesis of intermediate compounds 3, 4, and 5 as well as additional characterization of HBC–AOM GQDs can be found in the ESI (Fig. S1–S6†).

### 2.2. Synthesis and characterization of ITO NCs

Sn-doped In_2_O_3_ (ITO) nanocrystals were synthesized using a slow injection approach, already reported in the literature.^6^ Briefly, indium(iii) acetate and tin(iv) acetate were weighed in a 9 : 1 molar ratio, measuring 1 mmol in total. They were dispersed in 2 mL oleic acid (90%, technical grade) in a 50 mL, three-necked flask. The mixture was left to stir at 150 °C for 3 hours, under argon flux, to prepare the oleate precursors. In a 100 mL, three-necked flask, 13 mL oleyl alcohol (85%, technical grade) was heated to 150 °C, and left stirring under a nitrogen flux of 0.130 L min^−1^, to degas for 3 hours. Finally, after 3 hours the oleyl alcohol temperature was raised to 290 °C and the oleate mixture was injected into the flask using a syringe pump, at a 0.3 mL min^−1^ injection rate. After finishing the injection, the particles were left to grow for 15 minutes. Growth was then quenched by cooling the vessel rapidly with a mixture of compressed air and acetone. An ice bath was then used, at temperatures lower than 160 °C, to avoid the thermal shock shattering the glass flask.

The content from the flask was then collected in falcon tubes, and centrifuged twice at 6000 rcf for 10 minutes, adding ethanol to precipitate and hexane to re-disperse. The final colloid was stored in 3 mL of hexane, adding 10 μL oleic acid as a stabilizing agent.

All the chemicals were purchased from Sigma Aldrich and used as received. Metal acetates were stored in a nitrogen glovebox, and oleic acid was stored at 4 °C.

To characterize the obtained NCs, TEM imaging (Fig. S7a†), XRD (Fig. S7b†), ICP-OES analysis, and absorption spectroscopy (Fig. S8a,† black line) were carried out. The Sn doping level and NC solution concentration were determined by ICP-OES. The analysis revealed an Sn doping concentration in the In_2_O_3_ matrix of 10.9%.

### 2.3. Oxidation of HBC–AOM graphene quantum dots

Evidence for hole storage in HBC–AOM GQDs was provided *via* oxidative titration experiments by mixing a 12 μM solution of HBC–AOM in anhydrous chloroform (total volume of 3 mL) with increasing amounts of a 0.75 mM solution of 2,3,5,6-tetrafluoro-7,7,8,8-tetracyanoquinodimethane (F4TCNQ, Ossila) prepared in the same solvent. The amount of F4TCNQ added each time was chosen to match molar ratios between F4TCNQ and HBC–AOM of 0.5, 1, and 2. In order to prevent any dilution effects on the overall response, the amounts of F4TCNQ added to the HBC–AOM solution were low in volume: 23, 47, and 96 μL, respectively.

### 2.4. Functionalization of ITO NCs with HBC–AOM graphene quantum dots

A functionalized mixture of ITO NCs and HBC–AOM GQDs was obtained by mixing in a 10 mL vial (i) 10 μL of ITO NCs from a 9.1 mg mL^−1^ stock solution with (ii) 46 μL of HBC–AOM from a 1.7 mM freshly prepared stock solution and (iii) 1250 μL of anhydrous toluene. Under these conditions, the solution is characterized by a large excess of graphene quantum dots (2.53 : 1 weight ratio, 1.18 × 10^4^ molar ratio). To promote the interactions and functionalization, the vial was sealed and magnetically stirred in an Ar-filled glovebox at 400 rpm overnight and for an additional hour before each optical characterization experiment.

### 2.5. Optical measurements

Absorption spectra of HBC–AOM and HBC–AOM/F4TCNQ in anhydrous chloroform were recorded with a Varian Cary 5000 UV-Vis NIR spectrophotometer in the 250–1500 nm spectral range using a 10 mm optical path absorption cell (110 – Macro cells, Hellma). The photocharging dynamics of ITO NCs and the HBC–AOM/ITO NCs functionalized mixture was obtained by recording a temporal series of absorbance spectra during UV light exposure with a 10 seconds step between subsequent acquisitions. These experiments were performed in an Ar-filled glovebox (<0.1 ppm of O_2_ and H_2_O) using a portable fiber-coupled spectrometer (ASD LabSpec 4 Standard-Res Lab Analyzer, Malvern Panalytical) able to collect a full spectrum in the 350 nm to 2500 nm spectral range with an integration time of 5–6 s. Two low-OH optical fibers connect an external fiber optic illuminator source and the spectrometer to a four-light port cuvette holder (CVH100, Thorlabs, Inc). A CaF_2_ lens (LA5315, Thorlabs, Inc.) at the input port collimates the incoming light through a 4 mm optical path cell with 4 optical windows (114F – Semi-Micro cells, Hellma) that contains the sample under investigation dissolved in anhydrous toluene (typical concentration of ITO NCs ∼5 nM). A CaF_2_ lens at the output port refocuses the transmitted light intensity into the second optical fiber connecting the cuvette holder to the spectrometer. UV light illumination is provided by a deep UV LED (M300L4, nominal wavelength: 300 nm, bandwidth: 20 nm, Thorlabs, Inc) controlled by a power supply driver (LEDD1B, Thorlabs, Inc). The LED is directly mounted on the cuvette holder *via* a 1-inch length lens tube spacer (SM1S10, Thorlabs, Inc.). The UV light illumination direction is orthogonal to the optical axis used for the absorbance measurements and is outside the spectral range of the spectrometers, and this does not affect the acquisition of the transmission/absorbance spectra. The extended UV transparency window of anhydrous toluene in the region covered by the UV LED spectra ensures the stability of the solvent during UV illumination allowing us to exclude any solvent decomposition that might affect the outcome of the photodoping measurements.

## 3. Results & discussion

### 3.1. Oxidation of HBC–AOM graphene quantum dots

**Fig. 1:**
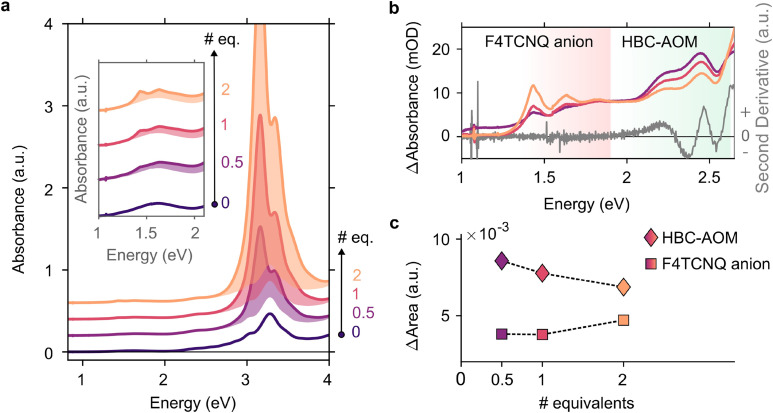
Oxidation of HBC–AOM graphene quantum dots induced by small additions of F4TCNQ molecules. (a) Absorbance spectra of the HBC–AOM/F4TCNQ mixture for increasing F4TCNQ equivalents (# equivalents are defined as the ratio between F4TCNQ and HBC–AOM mole number). Spectra in the inset for 0.5, 1, and 2 equivalents show the absorbance peaks associated with the F4TCNQ single anion. The shaded area in both the main panel and inset represents the net contribution of F4TCNQ addition. (b) Variation of HBC–AOM/F4TCNQ absorbance with respect to the un-doped HBC–AOM sample (dark violet spectrum in panel (a)) in the region of F4TCNQ anion and HBC–AOM optical bandgaps. Dark grey spectrum shows the second derivative of the HBC–AOM absorbance spectrum and the associated optically allowed transitions (negative peaks at 2.37 eV and 2.54 eV, respectively). Panel (c) shows the variation of the area below the spectra of panel (b) in the regions of F4TCNQ anion and HBC–AOM optical bandgaps for increasing F4TCNQ equivalents.

In the context of energy storage and conversion, graphene quantum dots are attracting increasing attention^16–19^ due to their peculiar optical and electronic properties as well as their low environmental impact and versatility in terms of functionalization with both electron withdrawing and donating functional groups.^20,21^ Precise control over their substitutional groups makes graphene quantum dots ideal candidates to address steady-state electron–hole separation in the presence of a heavily electron-doped counterpart, *i.e.* photodoped ITO NCs. First, to analyze the ability of HBC–AOM graphene quantum dots (Scheme 1) to act as the hole collector we prepared a 12 μM solution of HBC–AOM with small amounts of F4TCNQ. F4TCNQ with a concentration of 0.75 mM was employed to prevent the formation of a saturated solution and self-reduced anionic species.^22^ F4TCNQ, a 4-fold fluorinated derivative molecule of TCNQ, is a widely used strong molecular p-dopant.^23,24^ Depending on the relative position of the ionization energy of the targeted molecule and the electron affinity for the single and double reduction of F4TCNQ (5.2 and 4.7 eV, respectively^23^), this electron acceptor is able to extract either one or two electrons from the targeted molecule. The extraction of either one or two electrons is revealed by strong modifications of the optical absorption properties. These modifications provide a non-invasive optical tool to assess the level of oxidation of the targeted molecule. In particular, a single reduction of F4TCNQ leads to the formation of sub-bandgap NIR absorption features characterized by two prominent peaks at 1.45 and 1.65 eV, which are absent in the neutral (*i.e.* non-ionized) F4TCNQ molecule optical response. Double reduction instead leads to the formation of a single peak in the UV region centered around 3.7 eV.^23^Fig. 1a shows the evolution of the HBC–AOM absorbance spectra during the titration with increasing equivalents of F4TCNQ molecules. Subsequent additions lead to the formation of the F4TCNQ anion absorption peaks that are both clearly distinguishable in the region between 1 and 2 eV (inset of Fig. 1a). The formation of single F4TCNQ anion species is consistent with the HOMO energy level of HBC–AOM as obtained by DFT calculations (−5.13 eV *vs*. vacuum, Fig. S6†) which in absolute value is closer to the electron affinity value for single reduction (5.2 eV) than for double reduction (4.7 eV). As the double ionization of the F4TCNQ is energetically unfavorable, no absorbance peak in the UV region can be detected, which corresponds to the dianion. Along with the formation of the F4TCNQ anion, the spectra in Fig. 1a show that even after the first F4CTNQ addition a strong absorbance band located at 3.1 eV appears. This absorbance band, which is associated with the neutral F4TCNQ species suggests that only a small fraction of F4TCNQ molecules is reduced. A closer look at the relative absorbance spectra (Fig. 1b) calculated with respect to the un-doped HBC–AOM absorbance spectrum in the region of F4TCNQ anion and HBC–AOM optical bandgaps shows the presence of an isosbestic point located at 1.89 eV as a function of the increasing F4TCNQ equivalents. This suggests that the increase of F4TCNQ anion transition's oscillator strength (Fig. 1c) occurs at the expense of a reduction of HBC–AOM transition's oscillator strength, proving that F4TCNQ single reduction is inherently connected to the ability of HBC–AOM GQDs to permanently host one hole in their HOMO level.^25^

### 3.2. Photodoping of an ITO NC/HBC–AOM functionalized mixture

After assessing the possible use of HBC–AOM GQDs to host holes, we proceeded to investigate their role during the photodoping of ITO NCs. To this aim, we prepared a solution of HBC–AOM/ITO NCs. Taking into consideration the imbalance between the ability of ITO NCs to store more than hundreds of electrons upon photodoping, as evidenced by previous oxidative titration experiments,^6,7^ and of HBC–AOM to host one hole in the HOMO level, we prepared a mixture with a large excess of HBC–AOM GQDs, on the order of 10^4^ (molar ratio). Regarding the ITO NCs employed in this work, we made use of nanoparticles with a diameter of ∼13 nm and a tin doping level of ∼10.9% (Sn/Tot). The aliovalent Sn doping induces a strong localized surface plasmon resonance (LSPR)^26^ in the NIR range peaking at 0.74 eV (Fig. S8a,† black line) as a result of the presence of free electrons confined in a nanometric volume. By monitoring the absorption spectra through the optical setup previously described, we first observed that a simple solution obtained by mixing the HBC–AOM with ITO NCs (Fig. S8a,† red line) does not cause any appreciable variation neither in the spectral position of the LSPR peak (Fig. S8b†) nor in the spectral shape (Fig. S8c†). In contrast, prolonged stirring under anhydrous conditions leads to a significant modification of the absorbance spectrum of the mixture (Fig. S8a,† blue line). An overall decrease in both the LSPR peak and HBC–AOM absorbance and a small redshift of the LSPR (Fig. S8b,† blue line) point toward an effective functionalization of ITO NCs with HBC–AOM graphene quantum dots. In particular, the redshift of the LSPR is a clear indication that the ITO NC dielectric environment has changed due to the progressive adsorption of HBC–AOM.^27^ The absence of a substantial broadening of the LSPR in the functionalized mixture (Fig. S8c†) allows us also to exclude any effect due to the aggregation of the NCs. Moreover, this evidence precludes any strong hybridization between ITO NCs and HBC–AOM^28,29^ allowing us to analyze the optical behavior of the mixture in terms of the optical properties of its constituents.

**Fig. 2:**
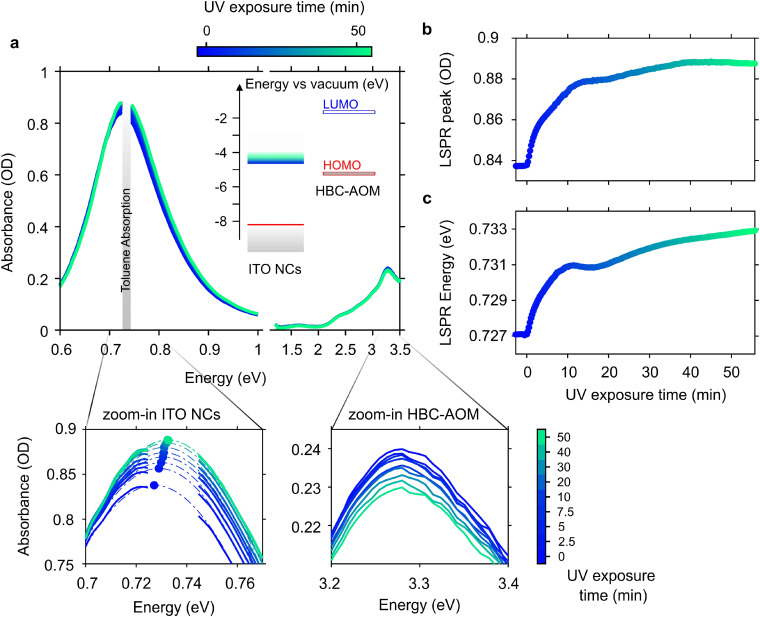
(a) Evolution of the absorbance spectra of the HBC–AOM/ITO NC functionalized mixture (2.53 : 1 weight ratio, 1.18 × 10^4^ molar ratio) upon UV light exposure. Time zero marks the start of UV light exposure. Inset shows the energy level alignment of ITO NC and HBC–AOM graphene quantum dots (the latter obtained *via* DFT calculations, Fig. S6†). Zoom-in panels highlight the variation of ITO NC LSPR and HBC–AOM peak absorbance at selected UV exposure times. Panels (b) and (c) show the evolution of the ITO NC LSPR peak absorbance and the LSPR peak energy upon UV light exposure, respectively. Both dynamics are obtained by fitting each spectrum in the LSPR region between 0.69 eV and 0.77 eV with a Gaussian function. Standard errors of the fitting routine are observed between the data point dimensions.

The photodoping process in doped metal oxide nanocrystals is usually described in terms of the spectral evolution of both the LSPR and bandgap upon UV light exposure. Fig. 2a shows the result of the ITO photodoping in the functionalized mixture of HBC–AOM/ITO NCs upon prolonged UV light exposure (∼57 min) along with the energy level alignment of ITO NCs and HBC–AOM. After the introduction of extra photo-electrons, the LSPR peak value of ITO NCs increases in intensity (Fig. 2b) and undergoes a shift to higher energies (Fig. 2c)^5^ until saturation is reached.^30^ In our case, saturation occurs after ∼40 minutes of UV light exposure, whereas the main variations (∼80% of the full LSPR peak dynamic) occur within the first 10 minutes of illumination. These time scales are compatible with previous literature reports for photodoped ZnO NCs in the presence of EtOH as sacrificial hole scavenger and are representative of the time required to accumulate free electrons following the quenching of the photogenerated holes in the ITO NC valence band.^30^ The absorbance spectra of HBC–AOM instead show a small decrease in their peak intensity on the order of 4%, which can be likely related to a combined effect of photobleaching of the graphene quantum dots due to prolonged UV exposure and a reduction in the transition's oscillator strength due to hole accumulation during the photodoping process. In this regard, it is worth noting that half of the HBC–AOM peak absorbance bleaching occurs during the first 10 minutes of UV illumination, that is, on the same time scale of ITO NC LSPR main variations (Fig. S9†).

**Fig. 3:**
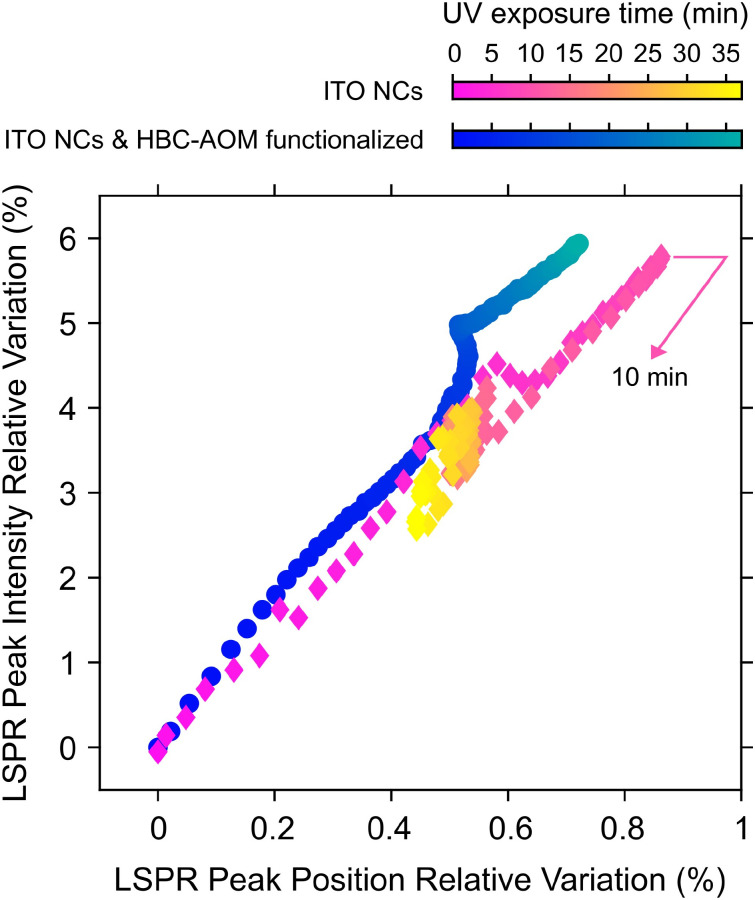
Characterization of the photodoping process of ITO NCs (magenta-to-yellow color palette) and the HBC–AOM/ITO NC functionalized mixture (blue-to-green palette) as a function of the UV exposure time. Relative variations of the ITO NC LSPR peak intensity (*y*-axis) and LSPR peak position (*x*-axis) are calculated with respect to the as-synthesized conditions. Values of the LSPR peak intensity and position are obtained by fitting each spectrum in the LSPR region with a Gaussian function. Integration time and waiting time between subsequent spectral acquisitions are common for both samples.

As discussed in ref. 30 the photodoping dynamics and the maximum number of photo-electrons that can be stored in each nanocrystal are strongly dependent on the nature of the hole scavenger. It is worth noting that photodoping of ITO NCs can still occur if the solution does not contain any sacrificial hole scavenger,^7^ suggesting that even the surrounding media (*e.g.* capping ligands and solvent) can act as a hole scavenger. However, as shown in Fig. 3, the absence of any hole scavenger or steady-state hole collector agent, such as HBC–AOM graphene quantum dots, affects the stability of the photo-doped electrons to prolonged UV exposure. Fig. 3, in fact, compares the LSPR relative variations of ITO NCs calculated upon photodoping, under two conditions: for the HBC–AOM/ITO NC functionalized mixture (data in Fig. 2) and for a solution of the same ITO NCs without HBC–AOM graphene quantum dots. A meaningful comparison between ITO NC LSPR dynamics is ensured by preparing the ITO NC solution without any variation of both ITO NCs concentration and total volume of solvent as well as by observing that the ITO NCs absorbance is not significantly modified around the UV LED spectra where ITO NCs and GQDs have an overlapping absorption region (Fig. S10†). The results we obtained demonstrate that the absence of graphene quantum dots precludes the possibility of maintaining over prolonged UV exposure a stationary state in which the maximum number of stored electrons per NCs is saturated. In particular, in contrast to what we observed in the case of the HBC–AOM/ITO NC functionalized mixture (blue-to-green palette in Fig. 3), the LSPR relative variation of ITO NCs (magenta-to-yellow palette) undergoes a decrease in its peak intensity and a redshift of its peak energy (yellow diamonds in Fig. 3 and spectra in Fig. S11†) after reaching its maximum values within the first 10 minutes of UV exposure, indicating the occurrence of irreversible photochemical processes in the ITO NCs solution.^30^ A comparison between LSPR normalized dynamics *vs*. UV exposure time (Fig. S12†) shows that the stability of the photodoped electrons is strongly enhanced in the HBC–AOM/ITO NC functionalized mixture from around 10 to nearly 40 minutes of UV exposure. Overall, these observations suggest that the ability of HBC–AOM graphene quantum dots to act as the hole collector supports the photodoping process of ITO NCs and stabilizes the photodoped electrons to prolonged UV exposure. As photodoping is a photochemical reduction process we can reason that the oxidation of HBC–AOM graphene quantum dots improves the stability of the photodoped electrons by competing with any other hole-quenching processes such as ligand and solvent oxidation. This further hole-quenching mechanism provides an additional oxidation channel to suppress detrimental hole-mediated multi-electron processes that would lead to a reduction in the number of photodoped electrons (such as non-radiative Auger recombination^31^). Considering the bleaching of the peak absorbance of HBC–AOM upon functionalization (Fig. S8a†) as indicative of the adsorption of the graphene quantum dots on the ITO NC surface and the further bleaching of HBC–AOM upon UV exposure (Fig. S9†), we can estimate that ∼30 electrons are transferred from the HOMO levels of graphene quantum dots to the valence band of photodoped ITO NCs (more details on the estimate can be found in the ESI†). Moreover, as the initial LSPR variations of both the HBC–AOM/ITO NC mixture and ITO NCs are similar during the first 2 minutes of UV exposure (which in Fig. 3 corresponds to an LSPR peak intensity relative variation reaching ∼1%), we can argue that the possible oxidation of HBC–AOM graphene quantum dots prevails over other oxidative processes once they are all completed. In this regard, the evidence for the almost complete photodoping of ITO NCs even in the functionalized mixture during the first minutes of UV exposure suggests that the limiting factor to the saturation behavior is primarily established by the oxidation of capping ligands and solvent rather than GQD oxidation. The combination of a kinetic process associated with ligand and solvent oxidation with slower kinetics representing the GQD oxidation might explain the apparent more efficient hole-scavenging role of ligands and solvents observed in the normalized LSPR peak dynamics in Fig. S12.† Finally, Fig. 3 and Fig. S12† allow us to qualitatively comment on the effect of ITO NC functionalization on the number of extra-electrons stored in the nanocrystals. Both figures show indeed that the ITO NC LSPR peak intensity reaches a common maximum value of +6% with respect to the as-prepared, un-photodoped conditions. Due to the proportionality between the LSPR peak and the number of stored electrons^32^ as well as the fact that the two samples have nominally the same ITO NC concentration we can safely conclude that the addition of GQDs does not significantly alter the number of electrons stored in the ITO NCs upon photodoping.

## 4. Conclusion

In summary, evidence for the stabilization of photodoped electrons is provided by comparing the photodoping process in ITO NCs and in a prototypical organic/inorganic functionalized mixture of HBC–AOM graphene quantum dots and ITO NCs. Comparative time-dependent analysis of absorbance spectra upon prolonged UV exposure reveals that saturation in the number of stored electrons is only reached when the reduction of photogenerated holes in ITO NCs is assisted by the oxidation of the graphene quantum dots. The results presented here highlight the role of a hole quencher in the photodoping process and support the implementation of metal oxide nanocrystals functionalized with electron-donating graphene quantum dots in the next generation of light-driven energy storage devices. As a further step, we foresee that the combination of ITO NCs with GQDs functionalized with proper redox mediators might enable the possibility of repeated and reversible photocharging.

## Data availability

Data supporting the study are available in the Zenodo repository under https://doi.org/10.5281/zenodo.8308892.

## Author contributions

A. Camellini and A. Rubino performed the HBC–AOM GQDs oxidation experiments. L. Rebecchi synthesized the ITO NCs and performed the structural characterization. A. Camellini, A. Rubino, and L. Rebecchi built the photodoping setup and performed the photodoping experiments. W. Niu, J. Ma, and X. Feng synthesized the HBC–AOM graphene quantum dots and performed the related structural characterization. S. W. Kim performed the optical characterization of HBC–AOM GQDs. A. Camellini and I. Kriegel conceptualized the experiment and wrote the manuscript with contributions from all other co-authors.

## Conflicts of interest

The authors declare no conflict of interest for the work herein reported.

## Supplementary Material

NR-015-D3NR03534D-s001
