# Supplementary material for: Improving the stability of photodoped metal oxide nanocrystals with electron donating graphene quantum dots
Source: Nanoscale. 2023 Sep 30;15(42):17138–46. doi: 10.1039/d3nr03534d (PMC10621319; doi:10.1039/d3nr03534d)
Supplement: NR-015-D3NR03534D-s001 [file NR-015-D3NR03534D-s001.pdf]

Electronic Supplementary Material (ESI) for Nanoscale.
This journal is © The Royal Society of Chemistry 2023

Supplementary Data

Improving the Stability of Photodoped Metal Oxide Nanocrystals with Electrons Donating Graphene Quantum Dots

Andrea Camellini,[a, b*] Luca Rebecchi,[a,c] Andrea Rubino,[a] Wenhui Niu,[d,e] Sang Won Kim,[f] Ji Ma,[d,e] Xinliang Feng, [d,e] Ilka Kriegel [a*]

[a-] *Functional Nanosystems, Istituto Italiano di Tecnologia (IIT), via Morego 30, 16163 Genova, Italy*

[b-] *Department of Mechanical Engineering, Columbia University, New York, New York 10027, USA*

[c-] *Dipartimento di Chimica e Chimica Industriale, Università degli Studi di Genova, Via Dodecaneso 31, 16146 Genova, Italy*

[d-] *Center for Advancing Electronics Dresden (cfaed) & Faculty of Chemistry and Food Chemistry, Technische Universität Dresden, 01062 Dresden, Germany*

[e-] *Max Planck Institute of Microstructure Physics, Weinberg 2, 06120 Halle, Germany*

[f-] *Samsung Advanced Institute of Technology (SAIT), Samsung Electronics Co. Ltd, Suwon, 16678, Republic of Korea.*

***Corresponding Author**

E-mail address: Andrea.Camellini@iit.it, Ilka.Kriegel@iit.it

Details on the synthesis of intermediate compounds 3, 4, 5 and HBC-AOM

- **General Methods and Materials**

All the reagents were obtained from Sigma Aldrich, TCI, abcr, BLD pharm, Strem, fluorochem, chempur. All these chemicals were used as received without further purification. All reactions dealing with air- or moisture-sensitive compounds were carried out in a dry reaction vessel under Ar atmosphere. Anhydrous dichloromethane and tetrahydrofuran were obtained from MBRAUN MB-SPS-5 solvent purification system. All the sensitive reactions were performed using standard vacuum-line and Schlenk techniques.

Thin layer chromatography (TLC) was performed on silica-coated aluminum sheets with a fluorescence indicator (TLC silica gel 60 F254, purchased from Merck KGaA).

Column chromatography was performed on silica (SiO_2, particle size 0.063-0.200 mm, purchased from VWR).

NMR spectra were recorded on a Bruker AV-II 300 spectrometer operating at 300 MHz for ^{1}H and at 75 MHz for ^{13}C at room temperature (23°C). CD_2Cl_2 ($\delta(^1H)$ = 5.33 ppm, $\delta(^{13}C)$ = 53.7 ppm) was used as solvents and as internal chemical shift reference. Chemical shifts (δ) are reported in ppm. The following abbreviations are used to describe peak patterns as appropriate: s = singlet, d = doublet, t = triplet, q = quartet, and m = multiplet.

Relative molar masses were determined by gel permeation chromatography (GPC) with an Aligent Technologies 1260 Infinity LC system equipped with two Resipore columns and RI and UV-vis detection. Chloroform was used as eluent with a flow rate of 1 mL min^{-1}. The measurements were carried out at 40 ºC. The molar masses were calculated relative to polystyrene standards with low dispersity.

The mass spectrometry analysis was performed on a Bruker Autoflex Speed MALDI TOF MS (Bruker Daltonics, Bremen, Germany) using trans-2-[3-(4-tert-butylphenyl)-2-methyl-2 propenylidene]malononitrile as matrix.

UV-visible spectra were measured on an Agilent Cary 5000 UV-vis-NIR spectrophotometer by using 10 mm optical-path quartz cell at room temperature.

Fluorescence spectra were recorded at room temperature on a PerkinElmer Fluorescence Spectrometer LS 55 using a 10 mm fluorescence quartz cell.

- **Synthesis of compound 3: 1,2-bis(4-(anthracen-9-yl)phenyl)ethyne**

In a 50 ml two-neck flask equipped with a condenser, compound **2** (1 g, 2.98 mmol), 9-anthraceneboronic acid (1.98 g, 8.93mmol) and K_2CO_3 (2.06 g, 13.6 mmol) were dissolved in 15 ml of dioxane, and 7 ml water then degassed by argon bubbling. $Pd(PPh_3)_4$ (344 mg, 0.30 mmol) was added into the solution. Then the reaction was stirred at 100 °C under argon overnight. Afterward, the reaction mixture was diluted with DCM (30 ml) and filtered. The mixture was washed three times with water, dried over sodium sulfate, and evaporated. The solid was purified by silica column chromatography (hexane/DCM = 4:1) to give compound **3** as a yellow solid (1.25 g, 79% yield).
HR-MS MALDI-TOF (m/z): calculated for $C_{42}H_{26}$ $[M]^+$ 530.2035; found: 530.2033. ^{1}H NMR (300 MHz, CD_2Cl_2) δ 8.55 (s, 2H), 8.09 (d, 8.4 Hz, 4H), 7.85 (d, 8.4 Hz, 4H), 7.71 (dq, 8.8, 1.0 Hz, 4H), 7.54-7.38 (m, 12H). ^{13}C NMR (75 MHz, CD_2Cl_2) δ 139.68, 136.74, 132.23, 132.08, 131.95, 130.60, 128.93, 127.40, 127.05, 126.17, 125.78, 123.04, 90.27.

- **Synthesis of compound 4: 9,9'-(3',4',5',6'-tetrakis(4-(anthracen-9-yl)phenyl)-[1,1':2',1''-terphenyl]-4,4''-diyl)dianthracene**

Compound **3** (500 mg, 0.94 mmol) was dissolved in dry 1, 4-dioxane (20 ml), and catalyst $Co_2(CO)_8$ (32.2 mg, 0.094 mmol) was added. The mixture was stirred at 110 °C under argon for 14 h. After cooling to room temperature, the solvent was removed under reduced pressure. The residue was washed by methanol and ethanol and to afford compound **4** as a white solid (410 mg, 82%).
HR-MS MALDI-TOF (m/z): calculated for $C_{126}H_{78}$ $[M]^+$ 1590.6104; found: 1590.5476.
NMR spectrum is not available due to bad solubility.

- **Synthesis of compound 5**

Compound **4** (100 mg, 63 μmol), compound **1** (263 mg, 1.26 mmol), and 10 ml anhydrous o-xylene were added into a 25 ml Schlenk flask; the mixture was bubbled with argon for 30 min and then heated to 150°C for 48 h. After that, the reaction mixture was cooled down to room temperature, and then the solvent was evaporated. The residue of the mixture was precipitated

with methanol and the crude product was collected by filtration. The crude product was further purified by recycle-GPC to remove the excess compound **1** and white target compound **5** (156 mg, 88%) was obtained.

^{1}H NMR (300 MHz, CD_2Cl_2) δ 7.90-6.61 (m, 72H), 4.81-4.56 (m, 6H), 3.96-3.66 (m, 6H), 3.47-3.15 (m, 12H), 2.99-2.80 (m, 6H), 1.44-1.36 (m, 12H), 1.27-1.02 (m, 60H), 0.80 (t, 7.2 Hz, 18H). ^{13}C NMR shows broad peaks due to the dynamic rotation of AOM groups.

FTIR: 2954.29, 2924.92, 2855.82, 2361.08, 2343.22, 1773.21, 1689.55, 1517.52, 1456.46, 1437.67, 1402.82, 1370.82, 1348.03, 1254.50, 1212.23, 1166.63, 1132.82, 1066.65, 1021.72, 955.32, 886.13, 826.29, 750.61, 722.37, 667.93, 659.89, 628.65, 582.00, 557.60 cm^{-1}.

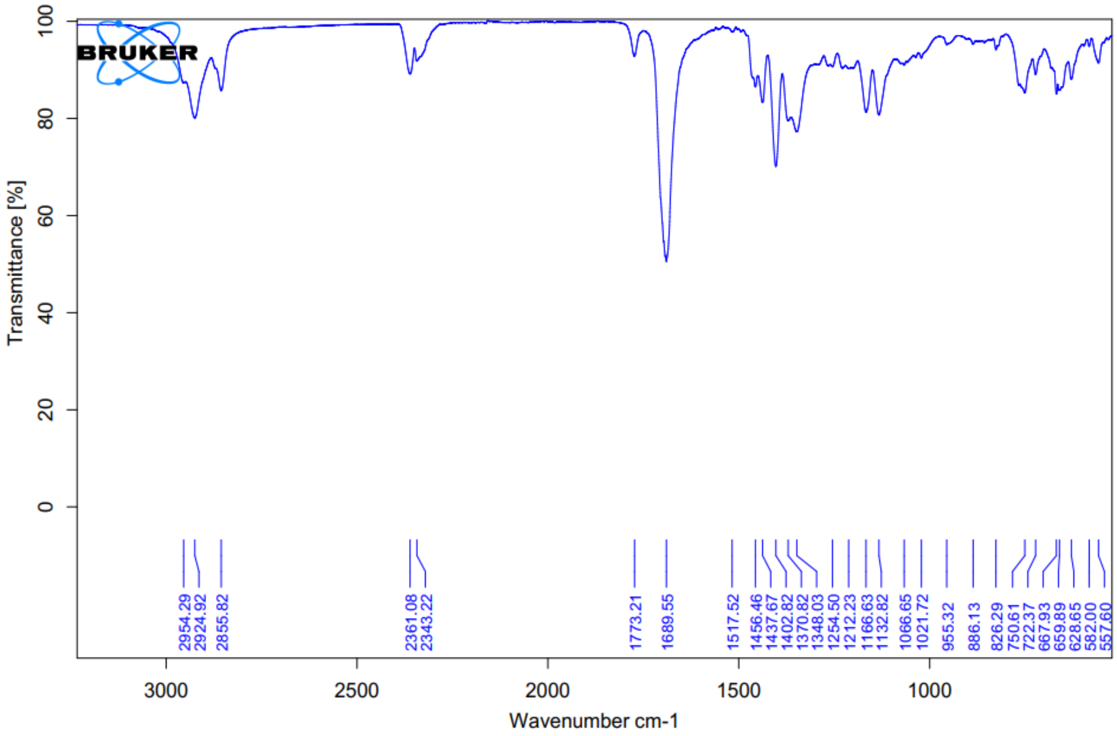

Figure S1 FTIR spectrum of compound **5**.

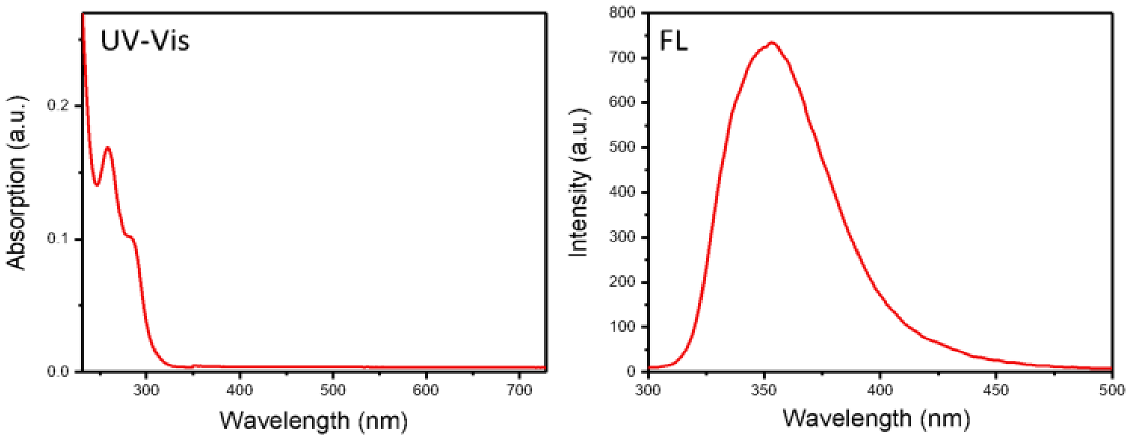

Figure S2 UV-Vis absorption and emission spectra of compound **5** in CH_2Cl_2.

- **Synthesis of HBC-AOM**

A solution of compound **5** (50 mg, 16.56 μmol) in dry DCM (50 ml) was degassed by argon bubbling for 30 min. A suspension of $FeCl_3$ (214 mg, 1.32 mmol) in nitromethane (1.2 ml) was added to the degassed solution. After stirring at room temperature for 5 h under continuous argon bubbling, the reaction was quenched by the addition of methanol to form brown precipitates. Filtration by suction using a membrane filter and washing intensively with methanol and water gave the target compound as a brown powder (44.8 mg, 90%).

Thanks to the introduction of bulky groups on the molecular periphery, the HBC-AOM showed excellent solubility in common organic solvents. The ^1H NMR spectrum shows very broad peaks in the aromatic region due to the aggregation in solution.

FTIR: 2925.62, 2855.25, 2349.60, 1774.08, 1693.08, 1517.56, 1457.13, 1437.77, 1401.98, 1370.10, 1348.87, 1254.83, 1198.69, 1166.65, 1133.17, 1062.18, 1033.79, 1021.64, 955.42, 886.16, 856.13, 826.14, 766.05, 750.39, 722.47, 650.92, 629.12 cm^{-1}

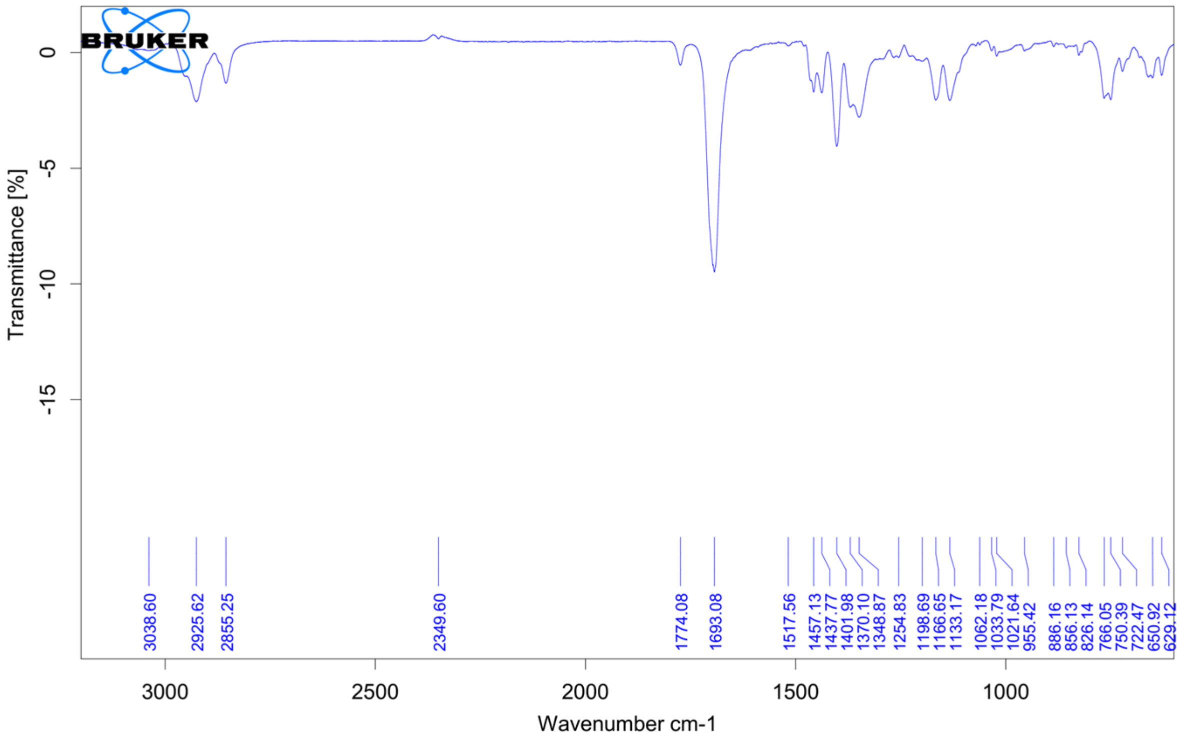

Figure S3 FTIR spectrum of HBC-AOM.

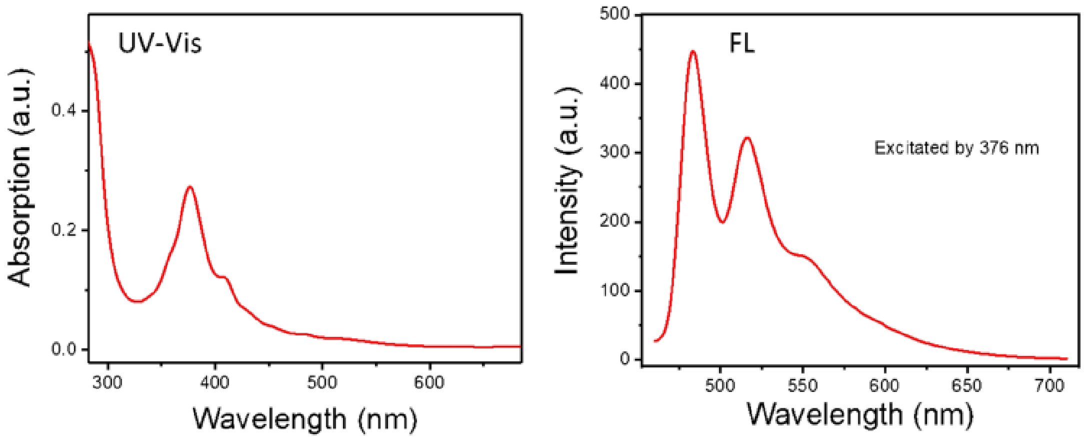

Figure S4 UV-Vis absorption and emission spectra of HBC-AOM in CH_2Cl_2.

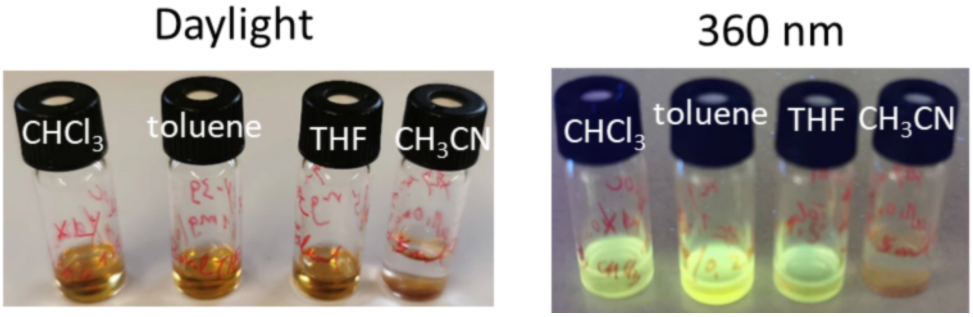

Figure S5 Photographs of the dispersions of HBC-AOM (5 mg/mL) in various organic solvents under daylight illumination and excited by 360nm.

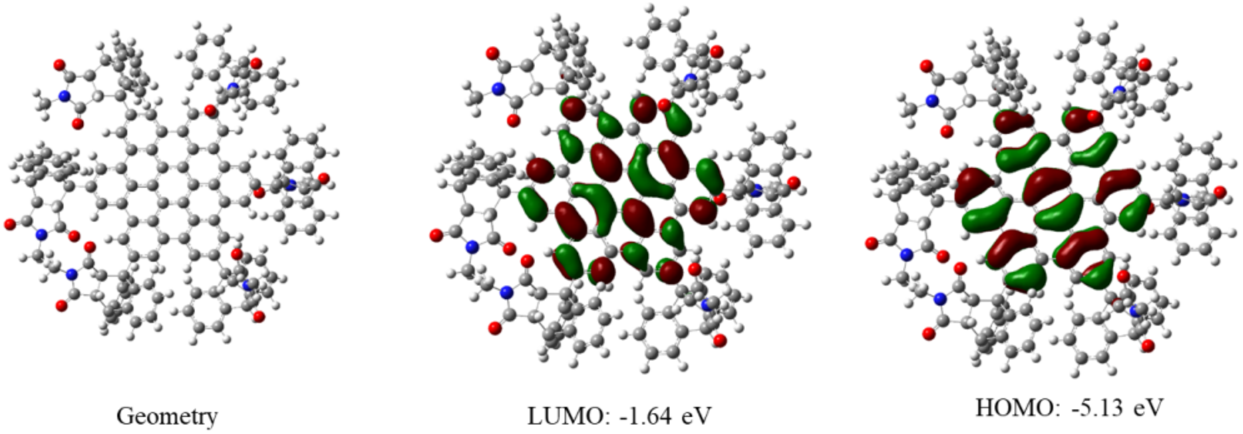

Figure S6. The optimized molecular geometry and frontier orbitals of HBC-AOM calculated by DFT using B3LYP/6-31G(d) basis set. The C_8H_{17} alkyl chains are replaced by methyl groups for clarity.

a b

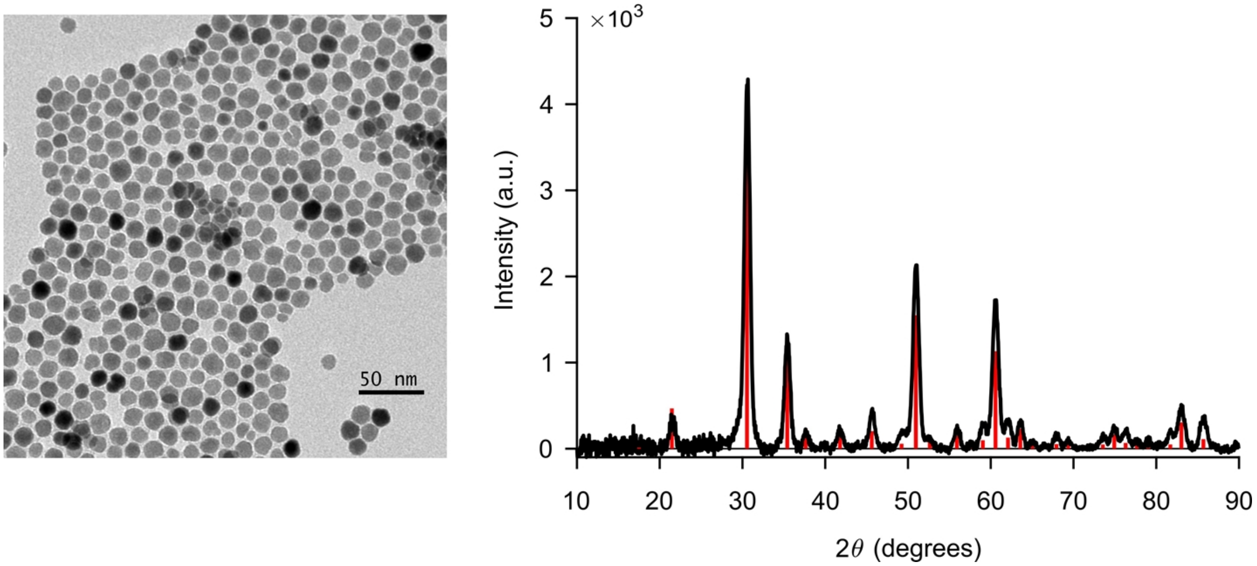

Figure S7 (a) TEM image and **(b)** X-ray diffraction pattern of ITO NCs used in this work. XRD diffraction pattern (black line) is overlapped to the normalized XRD reference pattern of ITO (red vertical lines, *ICSD 98-005-0849* card, ICDS Database).

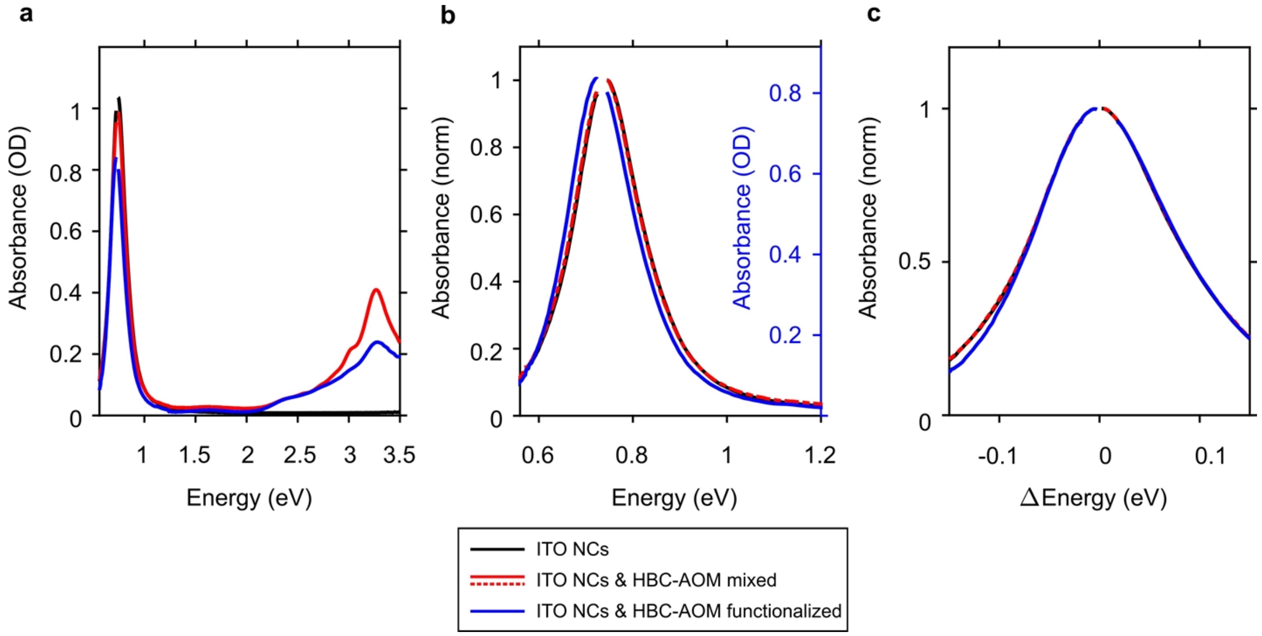

Figure S8 Absorbance spectra of HBC-AOM/ITO NCs (2.53:1 weight ratio, 1.18 10^4 molar ratio) upon simple mixture and functionalization (i.e. stirring overnight). Panel **(a)** shows the absorbance of ITO NCs (black line) along with HBC-AOM/ITO NCs mixture (red line) and HBC- AOM /ITO NCs functionalized mixture (blue line). **(b)** ITO NCs localized surface plasmon resonance (LSPR) of HBC- AOM/ITO NCs functionalized mixture shows a small redshift of 15.4 meV (35 nm) when compared to the LSPR-peak normalized absorbance spectra of ITO NCs and HBC-AOM/ITO NCs mixture. **(c)** Normalized and energy-shifted ITO NCs LSPR spectra show that neither simple mixture (red dashed line) nor functionalization (blue line) affects the as-synthesized ITO NCs LSPR spectral shape (black line).

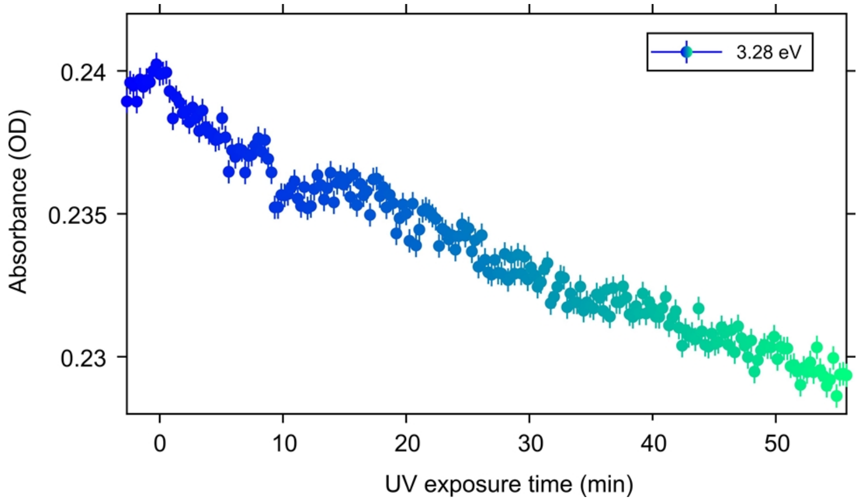

Figure S9 Evolution of the peak absorbance of HBC-AOM graphene quantum dots in the HBC-AOM/ITO NCs functionalized mixture upon UV light exposure. Error bars represent the standard deviation at the HBC-AOM peak absorbance (i.e. 3.28 eV) of ten subsequent spectra acquired without UV light exposure. The total decrease of the peak absorbance is ~4 % over 57 minutes of UV light exposure.

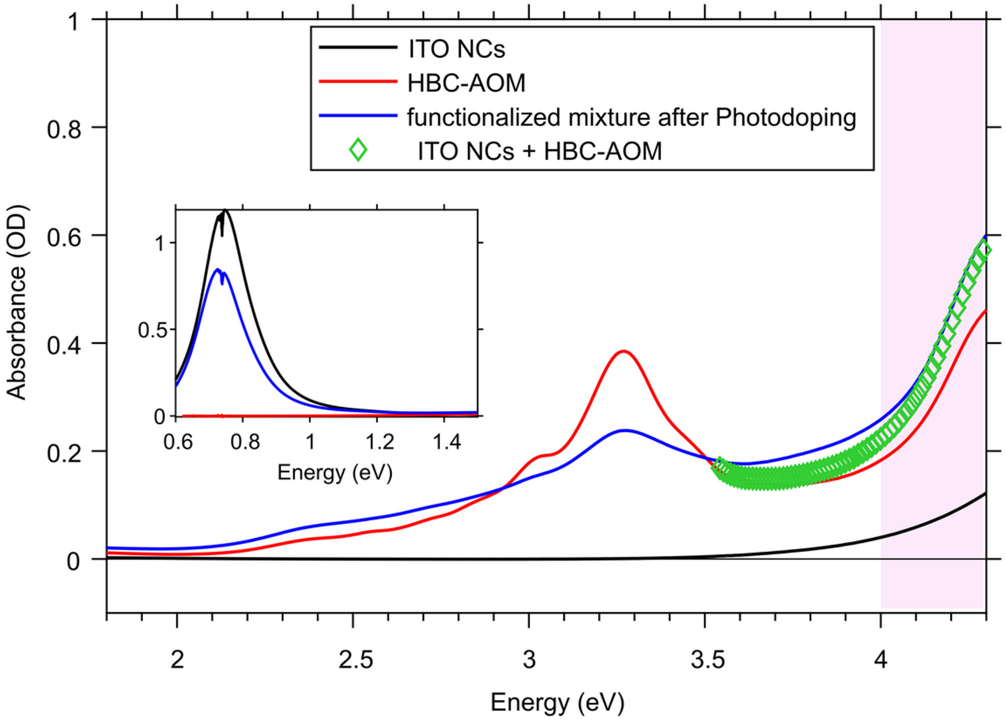

Figure S10 Comparison between absorbance spectra of ITO NCs, HBC-AOM GQDs, HBC-AOM GQDs/ITO NCs functionalized mixture and sum between ITO NCs absorbance and HBC-AOM GQDs absorbance – green empty diamonds. Absorbance spectra of ITO NCs and HBC-AOM GQDs are obtained by preparing two solutions having the same concentration of NCs and GQDs of the functionalized mixture (9.1 mg/mL and 5mg/mL in 1306μL of anhydrous toluene, respectively). The absorbance spectrum of the functionalized mixture was measured after the acquisition of the photodoping series in **Figure 2** of the manuscript. Violet shaded area represents the spectral region covered by the UV LED (300 nm central wavelength, 20 nm full width at half maximum). The inset shows the comparison between absorbance spectra in the regions of ITO NCs LSPR. All spectra are measured with a UV-Vis-NIR Varian Cary 5000 spectrophotometer.

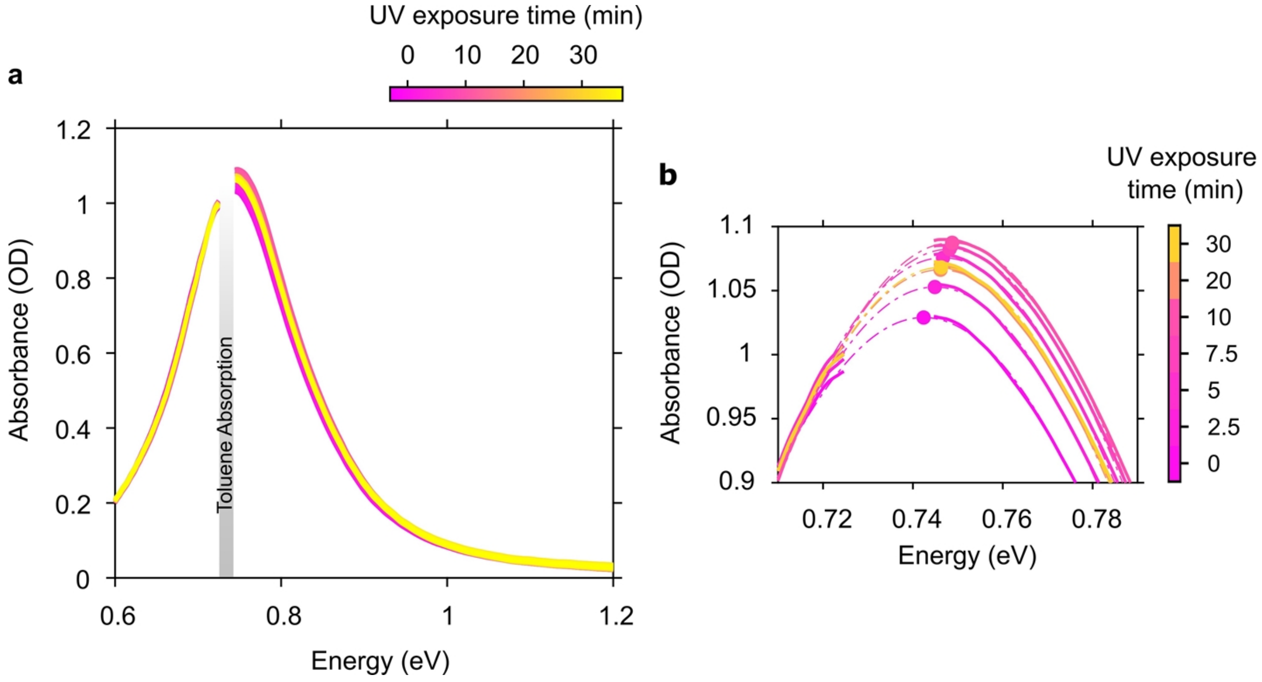

Figure S11 (a) Evolution of the absorbance spectra of ITO NCs upon UV light exposure. Time zero marks the start of UV light exposure. Panel **(b)** highlight the variation of the LSPR peak absorbance at selected UV exposure times.

Figure S12 Comparison between LSPR dynamics of ITO NCs and of a functionalized mixture of ITO NCs and HBC-AOM GQDs. **(a)** LSPR peak intensity and **(b)** LSPR peak energy *vs* UV exposure time. The dynamic in panel **(a)** and **(b)** are normalized with respect to LSPR peak intensity and energy of the as-prepare (i.e. un-photodoped) samples, respectively.

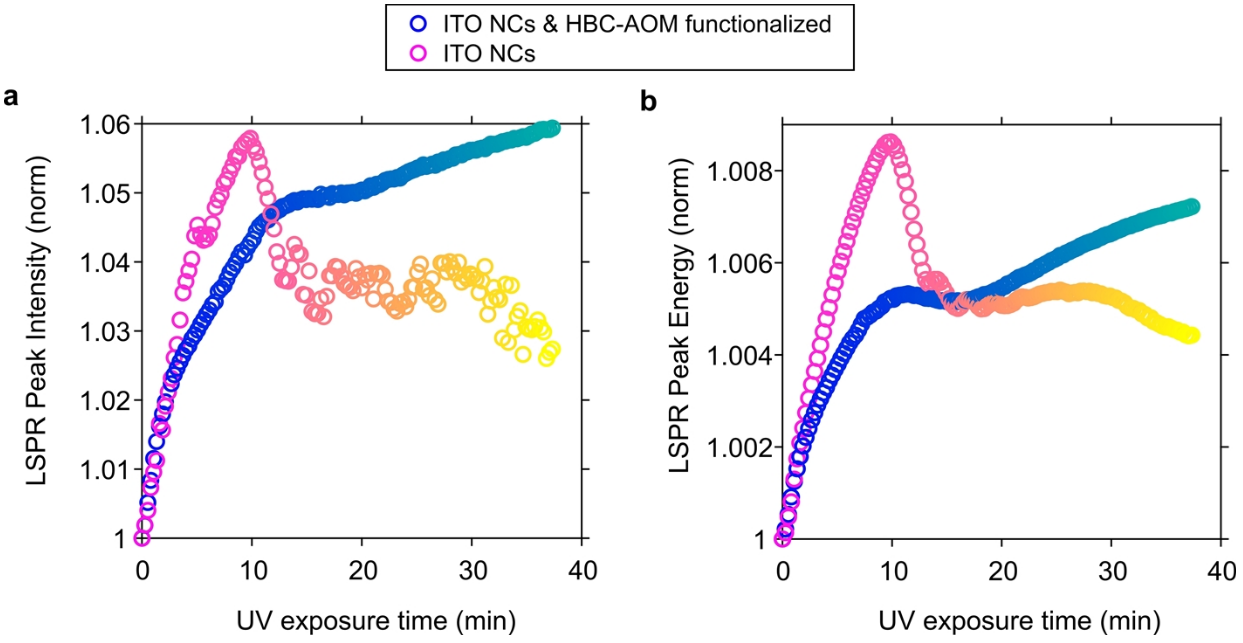

Numbers of electrons transferred from HBC-AOM to ITO NCs valence band upon photodoping

The number of electrons that are transferred from HOMO levels of HBC-AOM graphene quantum dots to the valence band of ITO NCs upon photodoping can be estimated as follow. Given the number of HBC-AOM graphene quantum dots in solution ($4.8\ 10^{16}$) and considering the bleaching of HBC-AOM peak absorbance (**Figure S8a**) we can calculate the number of graphene quantum dots that are absorbed on the ITO NCs surface as $\#GQD\ adsorbed\ = \#GQD \times \%GQD\ bleaching\ =\ 1.4\ 10^{16}$. Here, $\%GQD\ bleaching =\ 29.6\%$ represents the decrease of HBC-AOM peak absorbance in the functionalized HBC-AOM/ITO NCs mixture (**Figure S8a – red line**) with respect to the HBC-AOM/ITO NCs simple mixture (i.e. without prolonged stirring, **Figure S8a – blue line**). The number of adsorbed HBC-AOM graphene quantum dots per ITO NCs is therefore obtained as $\#GQD\ adsorbed/\#ITONCs\ =\ 3.4\ 10^{3}$ where $\#ITONCs$ is the number of ITO NCs in the solution obtained from ICP-OES mass spectroscopy ($4.15\ 10^{12}$). An estimate of the number of electrons transferred to the valence band of ITO NCs can thus be obtained by multiplying $\#GQD\ adsorbed$ per ITO NCs with the decrease of peak absorbance of HBC-AOM upon photodoping (**Figure S9**). As described in the main text almost half of this decrease occurs during the first 10 minutes of UV illumination, that is, on the same time scale of ITO NCs LSPR main variations (**Figure S8b**). By considering 1% as indicative of the HBC-AOM peak absorption decrease during the first 10 minutes of UV illumination we can obtain a conservative estimate of the number of electrons transferred to the valence band of ITO NCs as $\#GQD\ adsorbed/\#ITONCs\ \times\ 0.01\ =\ 34$. In this calculation, we considered that each

graphene quantum adsorbed onto the ITO NCs surface undergoes a single oxidation process (as detailed in **Figure 1** of the main text) and therefore transfers one electron from the HOMO level.
